# The genome sequence of the Argent & Sable moth, *Rheumaptera hastata* (Linnaeus, 1758) (Lepidoptera: Geometridae)

**DOI:** 10.12688/wellcomeopenres.26808.1

**Published:** 2026-06-23

**Authors:** Vlad Dincă, Marko Mutanen, Charlotte J. Wright, Joana I. Meier, Mark L. Blaxter, Wellcome Sanger Institute Tree of Life Management, Samples and Laboratory team, Wellcome Sanger Institute Scientific Operations: Sequencing Operations, Wellcome Sanger Institute Tree of Life Core Informatics team, Tree of Life Core Informatics collective, Project Psyche Community

**Affiliations:** 1 National Museum of Natural History “Grigore Antipa”, Bucharest, Romania; 2 University of Oulu, Oulu, Finland; 3 Tree of Life Programme, Wellcome Sanger Institute, Hinxton, England, UK

**Keywords:** Rheumaptera hastata, Argent & Sable, genome sequence, chromosomal, Lepidoptera

## Abstract

We present a genome assembly from an individual male *Rheumaptera hastata* (Argent & Sable; Arthropoda; Insecta; Lepidoptera; Geometridae). The assembly contains two haplotypes with total lengths of 589.77 megabases and 588.77 megabases. Most of haplotype 1 (99.85%) is scaffolded into 31 chromosomal pseudomolecules, including the Z sex chromosome. Haplotype 2 was assembled to scaffold level. The mitochondrial genome has also been assembled, with a length of 15.57 kilobases. This work is part of Project Psyche, a collaborative programme generating genomes for European butterflies and moths.

## Species taxonomy

Eukaryota; Opisthokonta; Metazoa; Eumetazoa; Bilateria; Protostomia; Ecdysozoa; Panarthropoda; Arthropoda; Mandibulata; Pancrustacea; Hexapoda; Insecta; Dicondylia; Pterygota; Neoptera; Endopterygota; Amphiesmenoptera; Lepidoptera; Glossata; Neolepidoptera; Heteroneura; Ditrysia; Obtectomera; Geometroidea; Geometridae; Larentiinae; *Rheumaptera*; *Rheumaptera hastata* (Linnaeus, 1758) (NCBI:txid691601).

## Background

**Figure 1.**
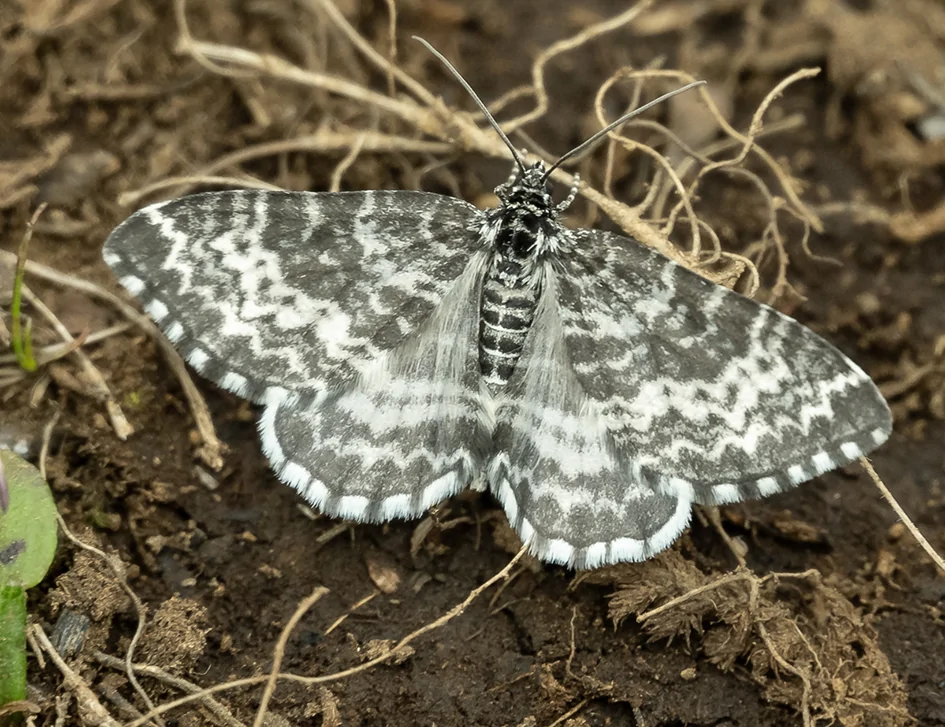
Image of *Rheumaptera hastata* ssp. *thulearia* from near Laugarfell, Iceland. Photograph by Pjt56.

*Rheumaptera hastata* (Linnaeus, 1758) ( Figure 1) is a Holarctic geometrid moth with a wide European distribution, largely absent from Mediterranean lowlands but extending north of the Arctic Circle and occurring through montane regions, including the central Alps (GBIF Secretariat, 2025; Wagner, 2025). It occupies bogs, blueberry-rich moist mountain forests and alpine dwarf-shrub belts. The larvae live in spun leaf ‘bags’ on *Vaccinium* spp. and *Rhododendron* spp., with additional use of *Betula* and *Salix*, and, in some Atlantic–boreal sites, *Myrica gale* ( Wagner, 2025; Wigglesworth *et al.*, 2007). Adults fly from May to July, depending on altitude, and larvae are typically found from late June to September ( Wagner, 2025). In parts of Scandinavia and the Alps it can be locally frequent, but elsewhere habitat loss (especially of bogs) and homogenised production forestry are noted pressures ( Wagner, 2025; Wigglesworth *et al.*, 2007).

We present a chromosome-level, haplotype-resolved genome sequence of the Argent & Sable, *Rheumaptera hastata*, sequenced as part of Project Psyche ( Wright *et al.*, 2025). The sequence data were derived from a male specimen collected from Otravaara, North Karelia, Finland.

## Methods

### Sample acquisition

The specimen used for genome sequencing was an adult male *Rheumaptera hastata* (specimen ID SAN28000250, ToLID ilRheHast1), collected from Otravaara, North Karelia, Finland (latitude 61.8916, longitude 30.1237; elevation 110 m) on 2023-06-01. The specimen was collected and identified by Vlad Dincă (University of Oulu).

### Nucleic acid extraction

Protocols for high molecular weight (HMW) DNA extraction developed at the Wellcome Sanger Institute (WSI) Tree of Life Core Laboratory are available on protocols.io ( Howard *et al.*, 2025). The ilRheHast1 sample was weighed and triaged to determine the appropriate extraction protocol. Tissue from the whole organism (30 mg) was homogenised by powermashing using a PowerMasher II tissue disruptor. HMW DNA was extracted in the WSI Scientific Operations core using the Automated MagAttract v2 protocol. DNA was sheared into an average fragment size of 12–20 kb following the Megaruptor®3 for LI PacBio protocol. Sheared DNA was purified by automated SPRI (solid-phase reversible immobilisation). The concentration of the sheared and purified DNA was assessed using a Nanodrop spectrophotometer and Qubit Fluorometer using the Qubit dsDNA High Sensitivity Assay kit. Fragment size distribution was evaluated by running the sample on the FemtoPulse system. For this sample, the final post-shearing DNA had a Qubit concentration of 58.86 ng/μL and a yield of 2 766.42 ng, with a fragment size of 16.7 kb.

RNA was extracted from whole organism tissue of ilRheHast1 in the Tree of Life Laboratory at the WSI using the RNA Extraction: Automated MagMax™ *mir*Vana protocol. The RNA concentration was assessed using a Nanodrop spectrophotometer and a Qubit Fluorometer using the Qubit RNA Broad-Range Assay kit. Analysis of the integrity of the RNA was done using the Agilent RNA 6000 Pico Kit and Eukaryotic Total RNA assay.

### PacBio HiFi library preparation and sequencing

Library preparation and sequencing were performed at the WSI Scientific Operations core. Libraries were prepared using the SMRTbell Prep Kit 3.0 (Pacific Biosciences, California, USA), according to the manufacturer’s instructions. The kit includes reagents for end repair/A-tailing, adapter ligation, post-ligation SMRTbell bead clean-up, and nuclease treatment. Size selection and clean-up were performed using diluted AMPure PB beads (Pacific Biosciences). DNA concentration was quantified using a Qubit Fluorometer v4.0 (ThermoFisher Scientific) and the Qubit 1X dsDNA HS assay kit. Final library fragment size was assessed with the Agilent Femto Pulse Automated Pulsed Field CE Instrument (Agilent Technologies) using the gDNA 55 kb BAC analysis kit.

The sample was sequenced on a Revio instrument (Pacific Biosciences, California, USA). Prepared libraries selected for multiplexing were pooled based on genome size, library molarity, and the intended plex level. Primers were annealed and polymerases were bound to generate circularised complexes, following the manufacturer’s instructions. Complexes were purified using SMRTbell beads, diluted to the Revio loading concentration, and spiked with a Revio sequencing internal control. The pooled libraries were sequenced on a Revio 25 M Plex SMRT cell in a 4-plex run. SMRT Link software (Pacific Biosciences), a web-based workflow manager, was used to configure and monitor the run and to carry out primary and secondary data analysis.

Specimen details, sequencing platforms, and data yields are summarised in Table 1.

**Table 1.** Specimen and sequencing data for *Rheumaptera hastata* (BioProject PRJEB80082).

Platform	PacBio HiFi	Hi-C	RNA-seq
**ToLID**	ilRheHast1	ilRheHast1	ilRheHast1
**Specimen ID**	SAN28000250	SAN28000250	SAN28000250
**BioSample (source individual)**	SAMEA115575585	SAMEA115575585	SAMEA115575585
**BioSample (tissue)**	SAMEA115575613	SAMEA115575613	SAMEA115575613
**Tissue**	whole organism	whole organism	whole organism
**Instrument**	Revio	Illumina NovaSeq X	Illumina NovaSeq X
**Run accessions**	ERR13660093	ERR13670052	ERR14986715
**Read count total**	2.03 million reads	316.97 million read pairs	70.40 million read pairs
**Base count total**	20.56 Gb	95.72 Gb	21.26 Gb

### Hi-C

***Sample preparation and crosslinking***

The Hi-C sample was prepared from 20–50 mg of frozen tissue from the whole organism of the ilRheHast1 sample using the Arima-HiC v2 kit (Arima Genomics). Following the manufacturer’s instructions, tissue was fixed and DNA crosslinked using TC buffer to a final formaldehyde concentration of 2%. The tissue was homogenised using the Diagnocine Power Masher-II. Crosslinked DNA was digested with a restriction enzyme master mix, biotinylated, and ligated. Clean-up was performed with SPRISelect beads before library preparation. DNA concentration was measured with the Qubit Fluorometer (Thermo Fisher Scientific) and Qubit HS Assay Kit. The biotinylation percentage was estimated using the Arima-HiC v2 QC beads.

***Hi-C library preparation and sequencing***

Biotinylated DNA constructs were fragmented using a Covaris E220 sonicator and size selected to 400–600 bp using SPRISelect beads. DNA was enriched with Arima-HiC v2 kit Enrichment beads. End repair, A-tailing, and adapter ligation were carried out with the NEBNext Ultra II DNA Library Prep Kit (New England Biolabs), following a modified protocol where library preparation occurs while DNA remains bound to the Enrichment beads. Library amplification was performed using KAPA HiFi HotStart mix and a custom Unique Dual Index (UDI) barcode set (Integrated DNA Technologies). Depending on sample concentration and biotinylation percentage determined at the crosslinking stage, libraries were amplified with 10–16 PCR cycles. Post-PCR clean-up was performed with SPRISelect beads. Libraries were quantified using the AccuClear Ultra High Sensitivity dsDNA Standards Assay Kit (Biotium) and a FLUOstar Omega plate reader (BMG Labtech).

Prior to sequencing, libraries were normalised to 10 ng/μL. Normalised libraries were quantified again to create equimolar and/or weighted 2.8 nM pools. Pool concentrations were checked using the Agilent 4200 TapeStation (Agilent) with High Sensitivity D500 reagents before sequencing. Sequencing was performed using paired-end 150 bp reads on the Illumina NovaSeq X. Specimen details, sequencing platforms, and data yields are summarised in Table 1.

### RNA-seq library preparation and sequencing

Libraries were prepared using the NEBNext ^®^ Ultra™ II Directional RNA Library Prep Kit for Illumina (New England Biolabs), following the manufacturer’s instructions. Poly(A) mRNA in the total RNA solution was isolated using oligo (dT) beads, converted to cDNA, and uniquely indexed; 14 PCR cycles were performed. Libraries were size-selected to produce fragment lengths of 100–300 bp. Libraries were quantified, normalised, pooled to a final concentration of 2.8 nM, and diluted to 150 pM for loading. Sequencing was carried out on the Illumina NovaSeq X to generate 150-bp paired-end reads.

### Genome assembly

Prior to assembly of the PacBio HiFi reads, a database of *k*-mer counts ( *k* = 31) was generated from the filtered reads using FastK. GenomeScope2 ( Ranallo-Benavidez *et al.*, 2020) was used to analyse the *k*-mer frequency distributions, providing estimates of genome size, heterozygosity, and repeat content.

The HiFi reads were assembled using Hifiasm in Hi-C phasing mode ( Cheng *et al.*, 2021, 2022), producing two haplotypes. Hi-C reads ( Rao *et al.*, 2014) were mapped to the primary contigs using bwa-mem2 ( Vasimuddin *et al.*, 2019). Contigs were further scaffolded with Hi-C data in YaHS ( Zhou *et al.*, 2023), using the --break option for handling potential misassemblies. The scaffolded assemblies were evaluated using Gfastats ( Formenti *et al.*, 2022), BUSCO ( Manni *et al.*, 2021) and MERQURY.FK ( Rhie *et al.*, 2020). The mitochondrial genome was assembled using MitoHiFi ( Uliano-Silva *et al.*, 2023), which runs MitoFinder ( Allio *et al.*, 2020) and uses these annotations to select the final mitochondrial contig and to ensure the general quality of the sequence.

### Assembly curation

The assembly was decontaminated using the Assembly Screen for Cobionts and Contaminants ( ASCC) pipeline. TreeVal was used to generate the flat files and maps for use in curation. Manual curation was conducted primarily in PretextView and HiGlass ( Kerpedjiev *et al.*, 2018). Scaffolds were visually inspected and corrected as described by Howe *et al*. (2021). Manual corrections included 12 breaks and 47 joins. This reduced the scaffold count by 25.3% and increased scaffold N50 by 0.8%. The curation process is described at https://gitlab.com/wtsi-grit/rapid-curation . PretextSnapshot was used to generate a Hi-C contact map of the final assembly.

### Assembly quality assessment

The Merqury.FK tool ( Rhie *et al.*, 2020), run in a Singularity container ( Kurtzer *et al.*, 2017), was used to evaluate *k*-mer completeness and assembly quality for both haplotypes using the *k*-mer database ( *k* = 31) computed prior to genome assembly. The analysis outputs included assembly QV scores and completeness statistics.

The genome was analysed using the BlobToolKit pipeline, a Nextflow ( Di Tommaso *et al.*, 2017) implementation of the earlier Snakemake version ( Challis *et al.*, 2020). The pipeline aligns PacBio reads using minimap2 ( Li, 2018) and SAMtools ( Danecek *et al.*, 2021) to generate coverage tracks. It runs BUSCO ( Manni *et al.*, 2021) using lineages identified from the NCBI Taxonomy ( Schoch *et al.*, 2020). For the three domain-level lineages, BUSCO genes are aligned to the UniProt Reference Proteomes database ( Bateman *et al.*, 2023) using DIAMOND blastp ( Buchfink *et al.*, 2021). The genome is divided into chunks based on the density of BUSCO genes from the closest taxonomic lineage, and each chunk is aligned to the UniProt Reference Proteomes database with DIAMOND blastx. Sequences without hits are chunked using seqtk and aligned to the NT database with blastn ( Altschul *et al.*, 1990). The BlobToolKit suite consolidates all outputs into a blobdir for visualisation. The BlobToolKit pipeline was developed using nf-core tooling ( Ewels *et al.*, 2020) and MultiQC ( Ewels *et al.*, 2016), with containerisation through Docker ( Merkel, 2014) and Singularity ( Kurtzer *et al.*, 2017).

We used lep_busco_painter to paint Merian elements along chromosomes ( Wright *et al.*, 2024). Merian elements represent the 32 ancestral linkage groups in Lepidoptera. The painting process utilised BUSCO gene locations from the lepidoptera_odb10 set ( Kriventseva *et al.*, 2019) and chromosome lengths from NCBI Datasets. Each complete BUSCO gene (both single-copy and duplicated) was assigned to a Merian element based on a reference database, then plotted along chromosomes drawn to scale.

## Genome sequence report

### Sequence data

**Figure 2.**
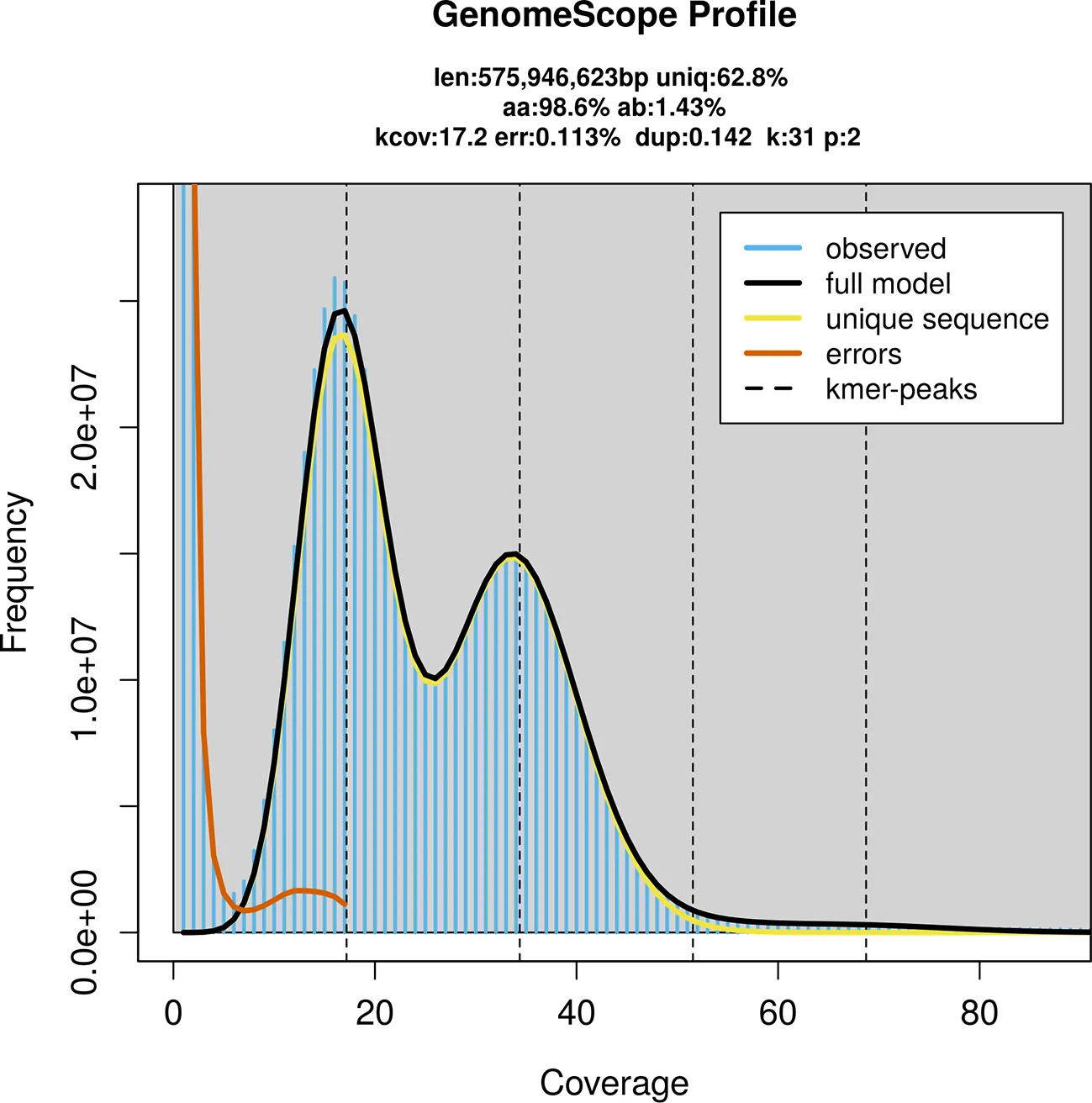
Frequency distribution of *k*-mers generated using GenomeScope2. The plot shows observed and modelled *k*-mer spectra, providing estimates of genome size, heterozygosity, and repeat content based on unassembled sequencing reads.

PacBio sequencing of the *Rheumaptera hastata* specimen generated 20.56 Gb (gigabases) from 2.03 million reads, which were used to assemble the genome. GenomeScope2.0 analysis estimated the haploid genome size at 576.64 Mb, with a heterozygosity of 1.43% and repeat content of 37.18% ( Figure 2). These estimates guided expectations for the assembly. Based on the estimated genome size, the sequencing data provided approximately 34× coverage. Hi-C sequencing produced 95.72 Gb from 633.94 million reads, which were used to scaffold the assembly. RNA sequencing data were also generated and are available in public sequence repositories. Table 1 summarises the specimen and sequencing details.

### Assembly statistics

The genome was assembled into two haplotypes using Hi-C phasing. Haplotype 1 was curated to chromosome level, while haplotype 2 was assembled to scaffold level. The final assembly has a total length of 589.77 Mb in 58 scaffolds, with 142 gaps, and a scaffold N50 of 20.9 Mb ( Table 2).

**Table 2.** Genome assembly statistics for *Rheumaptera hastata.*

Genome assembly	Haplotype 1	Haplotype 2
**Assembly name**	ilRheHast1.hap1.1	ilRheHast1.hap2.1
**Assembly accession**	GCA_964273775.1	GCA_964273725.1
**Assembly level**	chromosome	scaffold
**Span (Mb)**	589.77	588.77
**Number of chromosomes**	31	-
**Number of contigs**	200	196
**Contig N50**	6.74 Mb	7.12 Mb
**Number of scaffolds**	58	68
**Scaffold N50**	20.9 Mb	21.01 Mb
**Longest scaffold length (Mb)**	32.07	32.25
**Sex chromosomes**	Z	-
**Organelles**	Mitochondrial genome: 15.57 kb	-

**Figure 3.**
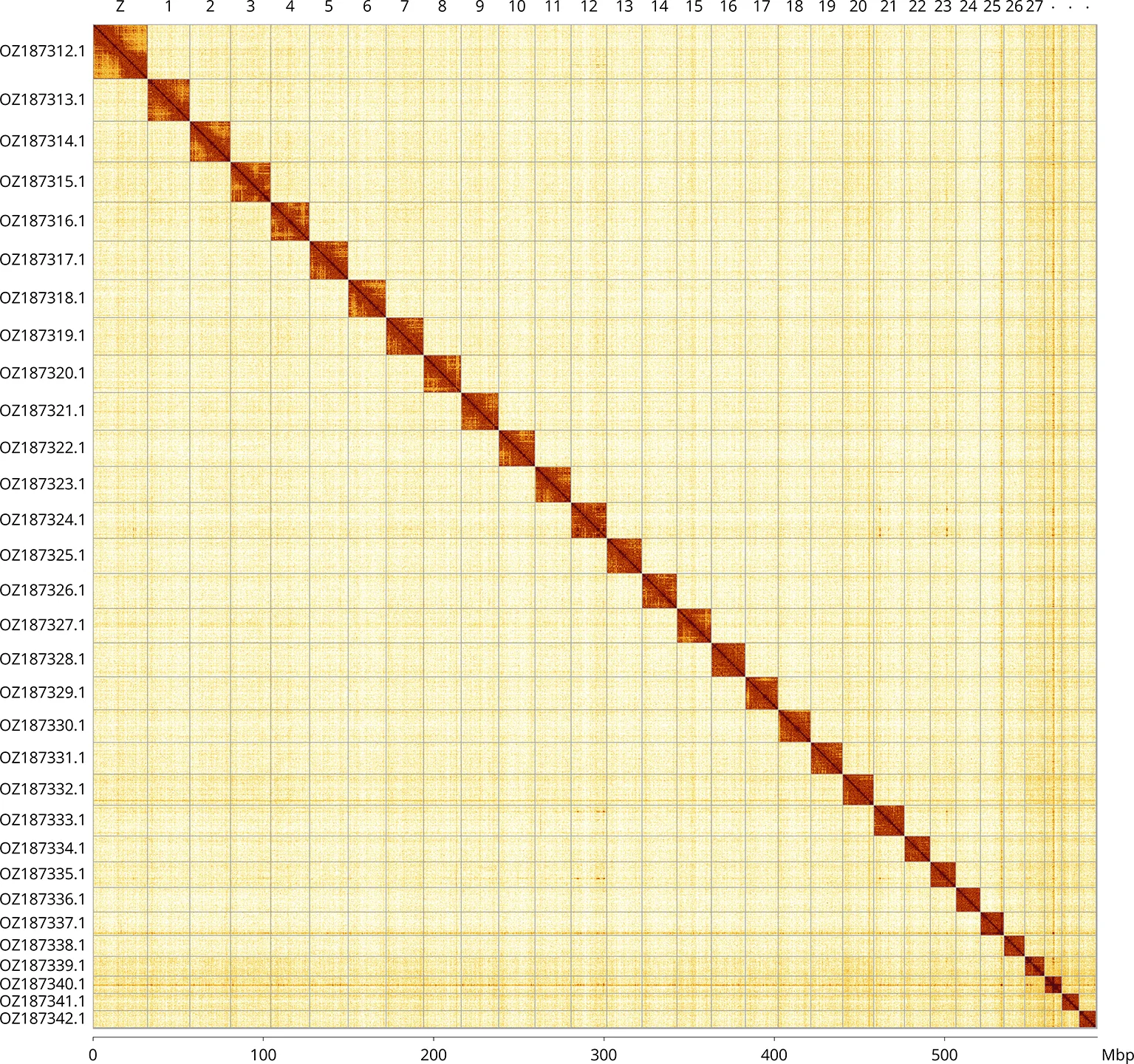
Hi-C contact map of the *Rheumaptera hastata* genome assembly. Assembled chromosomes are shown in order of size and labelled along the axes, with a megabase scale shown below. The plot was generated using PretextSnapshot.

**Figure 4.**
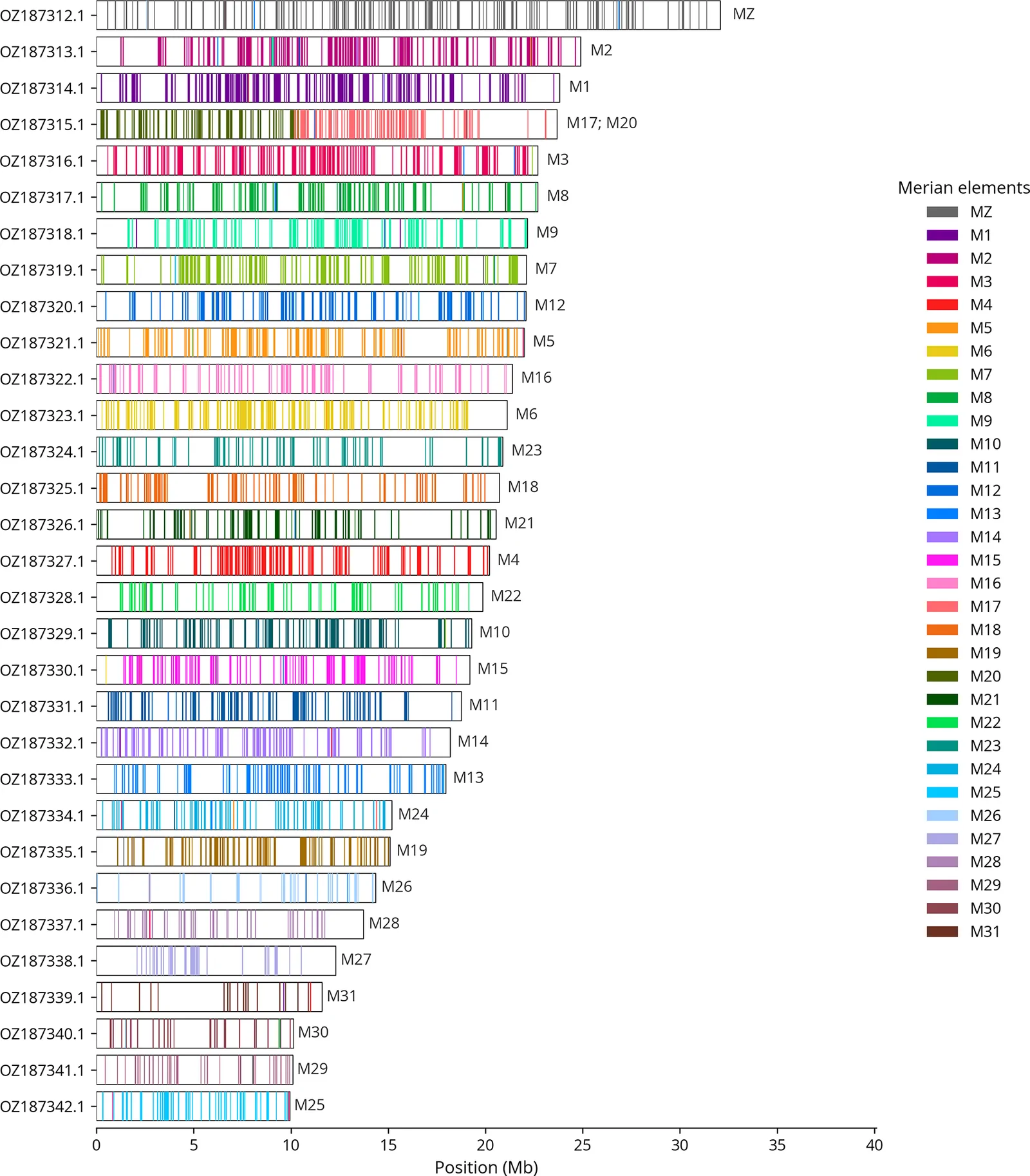
Merian elements painted across chromosomes in the ilRheHast1.hap1.1 assembly of *Rheumaptera hastata.* Chromosomes are drawn to scale, with the positions of orthologues shown as coloured bars. Each orthologue is coloured by the Merian element that it belongs to. All orthologues which could be assigned to Merian elements are shown.

Most of the assembly sequence (99.85%) was assigned to 31 chromosomal-level scaffolds, representing 30 autosomes and the Z sex chromosome. These chromosome-level scaffolds, confirmed by Hi-C data, are named according to size ( Figure 3; Table 3). Chromosome painting with Merian elements illustrates the distribution of orthologues along chromosomes and highlights patterns of chromosomal evolution relative to Lepidopteran ancestral linkage groups ( Figure 4). The Z chromosome was identified through synteny analysis with *Rheumaptera undulata* (GCA_964235345.1). During curation we note that contigs in the following regions have uncertain order and orientation: chromosome 28 (4.8–5.5 Mbp) and chromosome 25 (12.1–12.7 Mbp). The mitochondrial genome was also assembled (length 15.57 kb, OZ187343.1). This sequence is included as a contig in the multifasta file of the genome submission and as a standalone record.

**Table 3.** Chromosomal pseudomolecules in the haplotype 1 genome assembly of *Rheumaptera hastata* ilRheHast1.

INSDC accession	Molecule	Length (Mb)	GC%	Assigned Merian elements
OZ187313.1	1	24.90	37.50	M2
OZ187314.1	2	23.81	37.50	M1
OZ187315.1	3	23.69	37.50	M17;M20
OZ187316.1	4	22.71	37.50	M3
OZ187317.1	5	22.70	37.50	M8
OZ187318.1	6	22.16	37	M9
OZ187319.1	7	22.10	37.50	M7
OZ187320.1	8	22.08	37	M12
OZ187321.1	9	21.99	37	M5
OZ187322.1	10	21.39	37	M16
OZ187323.1	11	21.12	37	M6
OZ187324.1	12	20.90	37.50	M23
OZ187325.1	13	20.73	37	M18
OZ187326.1	14	20.56	37.50	M21
OZ187327.1	15	20.20	37.50	M4
OZ187328.1	16	19.86	37.50	M22
OZ187329.1	17	19.30	37.50	M10
OZ187330.1	18	19.20	37.50	M15
OZ187331.1	19	18.77	37.50	M11
OZ187332.1	20	18.19	38	M14
OZ187333.1	21	17.97	37.50	M13
OZ187334.1	22	15.20	37.50	M24
OZ187335.1	23	15.11	38	M19
OZ187336.1	24	14.36	37.50	M26
OZ187337.1	25	13.73	37.50	M28
OZ187338.1	26	12.31	37.50	M27
OZ187339.1	27	11.60	39	M31
OZ187340.1	28	10.14	41	M30
OZ187341.1	29	10.10	38.50	M29
OZ187342.1	30	9.96	38.50	M25
OZ187312.1	Z	32.07	37.50	MZ

### Assembly quality metrics

**Figure 5.**
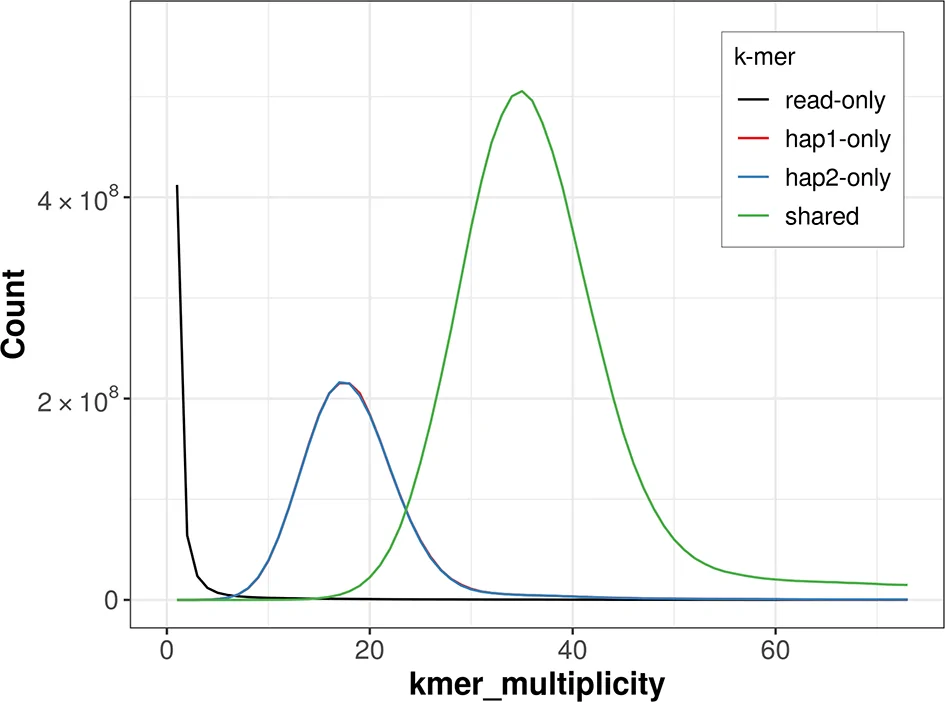
Evaluation of *k*-mer completeness using MerquryFK. This plot illustrates the recovery of *k*-mers from the original read data in the final assemblies. The horizontal axis represents *k*-mer multiplicity, and the vertical axis shows the number of *k*-mers. The black curve represents *k*-mers that appear in the reads but are not assembled. The green curve (the homozygous peak) corresponds to *k*-mers shared by both haplotypes and the red and blue curves (the heterozygous peaks) show *k*-mers found only in one of the haplotypes.

For haplotype 1, the estimated QV is 66.5, and for haplotype 2, 67.7. When the two haplotypes are combined, the assembly achieves an estimated QV of 67.1. The *k*-mer completeness is 73.43% for haplotype 1, 73.36% for haplotype 2, and 99.50% for the combined haplotypes ( Figure 5).

**Figure 6.**
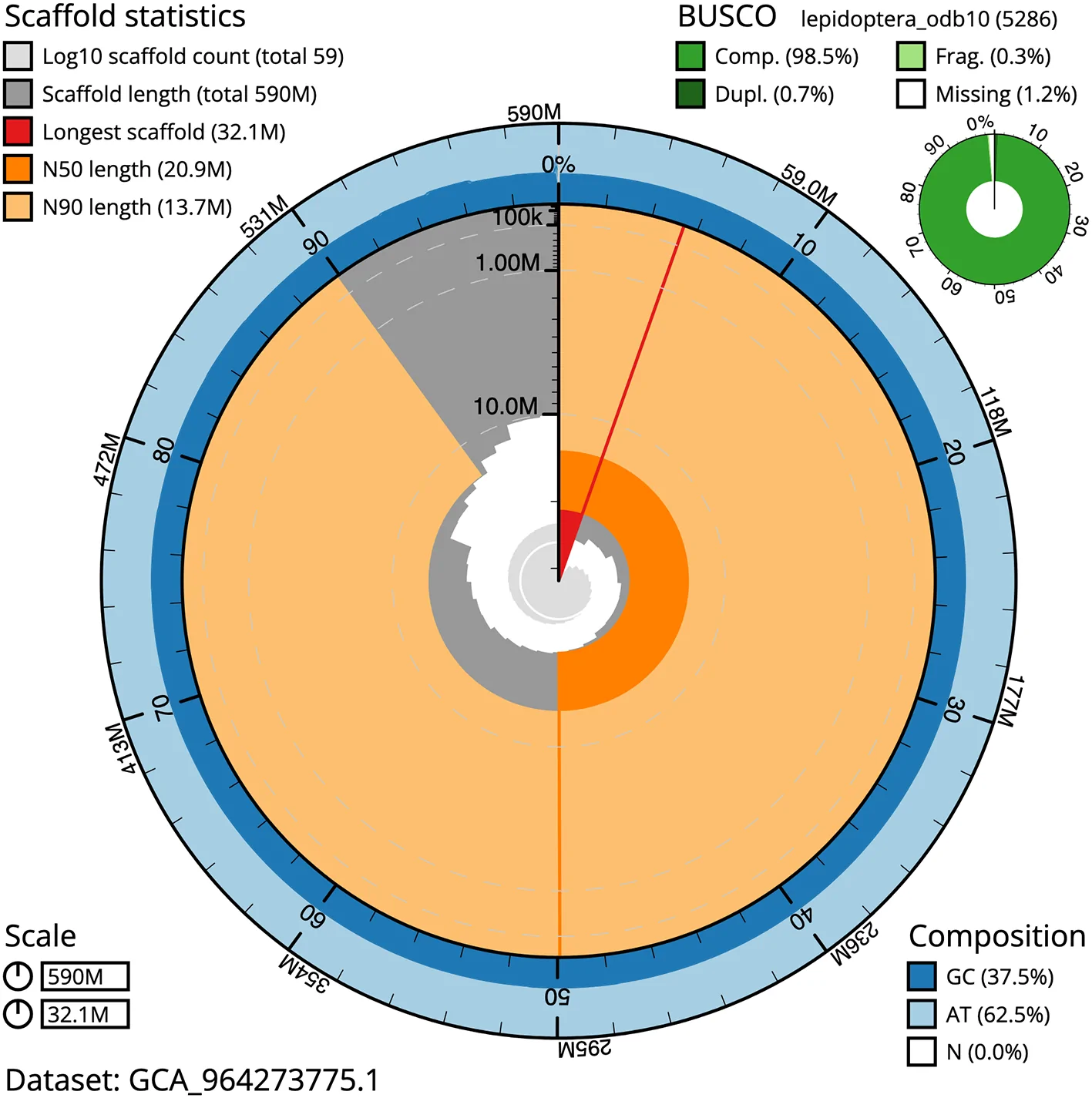
Assembly metrics for ilRheHast1.hap1.1. The BlobToolKit snail plot provides an overview of assembly metrics and BUSCO gene completeness. The circumference represents the length of the whole genome sequence, and the main plot is divided into 1,000 bins around the circumference. The outermost blue tracks display the distribution of GC, AT, and N percentages across the bins. Scaffolds are arranged clockwise from longest to shortest and are depicted in dark grey. The longest scaffold is indicated by the red arc, and the deeper orange and pale orange arcs represent the N50 and N90 lengths. A light grey spiral at the centre shows the cumulative scaffold count on a logarithmic scale. A summary of complete, fragmented, duplicated, and missing BUSCO genes in the set is presented at the top right. An interactive version of this figure can be accessed on the BlobToolKit viewer.

**Figure 7.**
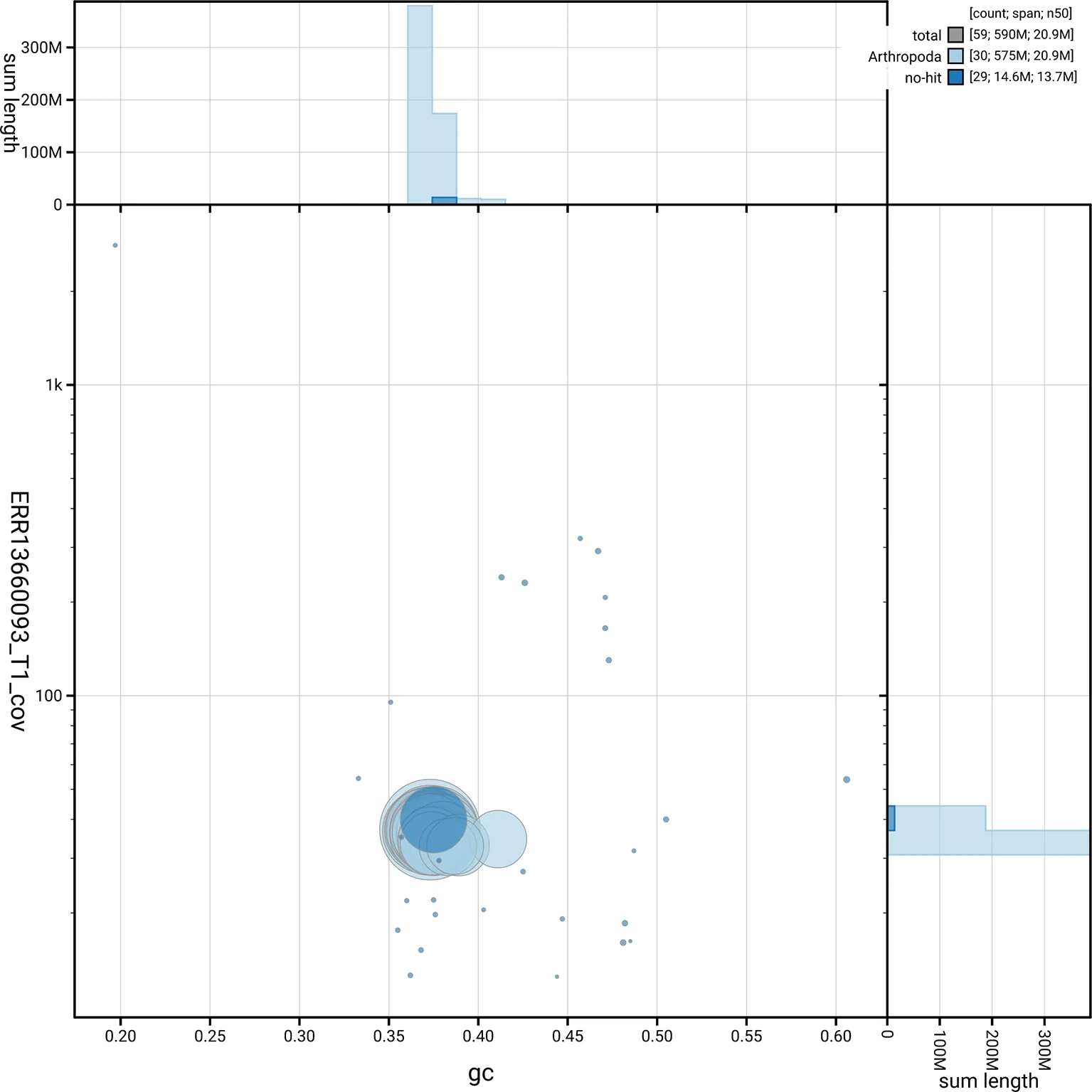
BlobToolKit GC-coverage plot for ilRheHast1.hap1.1. Blob plot showing sequence coverage (vertical axis) and GC content (horizontal axis). The circles represent scaffolds, with the size proportional to scaffold length and the colour representing phylum membership. The histograms along the axes display the total length of sequences distributed across different levels of coverage and GC content. An interactive version of this figure is available on the BlobToolKit viewer.

BUSCO analysis using the lepidoptera_odb10 reference set ( *n* = 5 286) identified 98.5% of the expected gene set (single = 97.9%, duplicated = 0.7%) in haplotype 1. For haplotype 2, BUSCO analysis identified 98.3% of the expected gene set (single = 97.7%, duplicated = 0.6%). The snail plot in Figure 6 summarises the scaffold length distribution and other assembly statistics for haplotype 1. The blob plot in Figure 7 shows the distribution of scaffolds by GC proportion and coverage for haplotype 1.

Table 4 lists the assembly metric benchmarks adapted from Rhie *et al.* (2021) and the Earth BioGenome Project Report on Assembly Standards September 2024. The EBP metric, calculated for the haplotype 1, is **6.C.Q66**, meeting the recommended reference standard.

**Table 4.** Earth Biogenome Project summary metrics for the *Rheumaptera hastata* assembly.

Measure	Value	Benchmark
EBP summary (haplotype 1)	6.C.Q66	6.C.Q40
Contig N50 length	6.74 Mb	≥ 1 Mb
Scaffold N50 length	20.90 Mb	= chromosome N50
Consensus quality (QV)	Haplotype 1: 66.5; haplotype 2: 67.7; combined: 67.1	≥ 40
*k*-mer completeness	Haplotype 1: 73.43%; Haplotype 2: 73.36%; combined: 99.50%	≥ 95%
BUSCO	Haplotype 1: C:98.5% [S:97.9%, D:0.7%], F:0.3%, M:1.2%, n:5 286; Haplotype 2: C:98.3% [S:97.7%, D:0.6%], F:0.4%, M:1.3%, n:5 286	S > 90%; D < 5%
Percentage of assembly assigned to chromosomes	99.86%	≥ 90%

**Table 5.** Software versions and sources used for *Rheumaptera hastata.*

Software	Version	Source
BLAST	2.14.0	ftp://ftp.ncbi.nlm.nih.gov/blast/executables/blast+/
BlobToolKit	4.3.9	https://github.com/blobtoolkit/blobtoolkit
BUSCO	5.5.0	https://gitlab.com/ezlab/busco
bwa-mem2	2.2.1	https://github.com/bwa-mem2/bwa-mem2
DIAMOND	2.1.8	https://github.com/bbuchfink/diamond
fasta_windows	0.2.4	https://github.com/tolkit/fasta_windows
FastK	1.1	https://github.com/thegenemyers/FASTK
GenomeScope2.0	2.0.1	https://github.com/tbenavi1/genomescope2.0
Gfastats	1.3.6	https://github.com/vgl-hub/gfastats
Hifiasm	0.19.8-r603	https://github.com/chhylp123/hifiasm
HiGlass	1.13.4	https://github.com/higlass/higlass
merian-busco-painter	v1.0.0	https://github.com/Karenvn/merian-busco-painter
MerquryFK	1.1.0-c1	https://github.com/thegenemyers/MERQURY.FK
Minimap2	2.24-r1122	https://github.com/lh3/minimap2
MitoHiFi	3	https://github.com/marcelauliano/MitoHiFi
MultiQC	1.14; 1.17 and 1.18	https://github.com/MultiQC/MultiQC
Nextflow	23.10.0	https://github.com/nextflow-io/nextflow
PretextSnapshot	0.0.5	https://github.com/sanger-tol/PretextSnapshot
PretextView	1.0.3	https://github.com/sanger-tol/PretextView
samtools	1.19.2	https://github.com/samtools/samtools
sanger-tol/ascc	0.1.0	https://github.com/sanger-tol/ascc
sanger-tol/blobtoolkit	0.6.0	https://github.com/sanger-tol/blobtoolkit
sanger-tol/curationpretext	1.4.2	https://github.com/sanger-tol/curationpretext
Seqtk	1.3	https://github.com/lh3/seqtk
Singularity	3.9.0	https://github.com/sylabs/singularity
TreeVal	1.4.0	https://github.com/sanger-tol/treeval
YaHS	1.2a.2	https://github.com/c-zhou/yahs

## Wellcome Sanger Institute – Legal and Governance

The materials that have contributed to this genome note have been supplied by a Tree of Life collaborator. The Wellcome Sanger Institute employs a process whereby due diligence is carried out proportionate to the nature of the materials themselves, and the circumstances under which they have been/are to be collected and provided for use. The purpose of this is to address and mitigate any potential legal and/or ethical implications of receipt and use of the materials as part of the research project, and to ensure that in doing so, we align with best practice wherever possible. The overarching areas of consideration are:

• Ethical review of provenance and sourcing of the material
• Legality of collection, transfer and use (national and international)

Each transfer of samples is undertaken according to a Research Collaboration Agreement or Material Transfer Agreement entered into by the Tree of Life collaborator, Genome Research Limited (operating as the Wellcome Sanger Institute), and in some circumstances, other Tree of Life collaborators.

## Data Availability

European Nucleotide Archive: Rheumaptera hastata. Accession number PRJEB80082; https://identifiers.org/ena.embl/PRJEB80082. The genome sequence is released openly for reuse. The *Rheumaptera hastata* genome sequencing initiative is part of the Sanger Institute Tree of Life Programme (PRJEB43745) and Project Psyche (PRJEB71705). All raw sequence data and the assembly have been deposited in INSDC databases. The genome will be annotated using available RNA-Seq data and presented through Ensembl at the European Bioinformatics Institute. Raw data and assembly accession identifiers are reported in Tables 1 and 2. Pipelines used for genome assembly at the WSI Tree of Life are available at https://pipelines.tol.sanger.ac.uk/pipelines. Table 5 lists software versions used in this study.
