# Supplementary material for: The genome sequence of the Argent & Sable moth, Rheumaptera hastata (Linnaeus, 1758) (Lepidoptera: Geometridae)
Source: Wellcome Open Res. 2026 Jun 23;11:370. [Version 1] doi: 10.12688/wellcomeopenres.26808.1 (PMC13591047; doi:10.12688/wellcomeopenres.26808.1)
Supplement: Peer review history

# Reviewer report: Reviewer response for version 1

**DOI:** 10.21956/wellcomeopenres.29518.r168361

**Published:** 2026-10-05

**Authors:** Laura Jimenez Burney

**Affiliations:** 1 University of Liverpool, Liverpool, UK

The authors present a very high-quality, chromosome-level genome assembly for *Rheumaptera hastata* using PacBio HiFi, Hi-C, and RNA-seq data. The assembly quality metrics (QV 66.5, 98.5% BUSCO completeness, and 31 pseudomolecules) are impressive and meet Earth BioGenome Project standards. 

 Minor comments and questions:

1. The authors mention that contig order and orientation remain uncertain on Chromosome 28 (4.8–5.5 Mbp) and Chromosome 25 (12.1–12.7 Mbp). Could you briefly comment on what might be causing this—for example, high repeat density, rDNA clusters, or local drops in Hi-C coverage?
2. The decontamination workflow using ASCC and BlobToolKit is clear, but many geometrid moths harbor bacterial endosymbionts like *Wolbachia *and *Spiroplasma*. Could you explicitly state whether any cobiont or endosymbiont sequences were detected and filtered out during screening, or if the sample was entirely free of non-eukaryotic reads?
3. GenomeScope2 estimates repeat content at ~37.2%. While full gene annotation will be delivered by Ensembl, a short summary sentence on the dominant repeat classes (or confirmation that repeat-masked FASTA files are available) would be very useful for researchers planning comparative or population genomic work.

Are sufficient details of methods and materials provided to allow replication by others?

Yes

Is the rationale for creating the dataset(s) clearly described?

Yes

Are the datasets clearly presented in a useable and accessible format?

Yes

Are the protocols appropriate and is the work technically sound?

Yes

Reviewer Expertise:

Population genetics applied to conservation. Special focus on cobionts such as Wolbachia and Spiroplasma in Lepidoptera.

I confirm that I have read this submission and believe that I have an appropriate level of expertise to confirm that it is of an acceptable scientific standard.

# Reviewer report: Reviewer response for version 1

**DOI:** 10.21956/wellcomeopenres.29518.r168358

**Published:** 2026-09-21

**Authors:** Jinmu Kim

**Affiliations:** 1 University of Illinois at Urbana-Champaign, Urbana, Illinois, USA

**Article assessment**

 The Data Note by Dinca V., et al. reports two haplotype assemblies of a male Rheumaptera hastata generated using PacBio HiFi, Hi-C, and RNA-seq data. Haplotype 1 is a high-quality 589.77 Mb chromosome-level assembly comprising 31 pseudomolecules with a QV of 66.5 and 98.5% BUSCO completeness. The Data Note is well written; however, a few minor comments should be addressed before the indexing.

**Detailed comments:**

 1. Figure 1: The author should provide the size of the specimen or include a scale bar in the figure.

 2. Table 1 lists “whole organism” as the tissue source for the PacBio, Hi-C, and RNA-seq datasets. However, the methods do not explain how the specimen was dissected among three different extraction processes. The authors should clarify whether all libraries were prepared from tissue aliquots obtained from the same individual or whether 3 individuals were used. For example, the nucleic acid extraction section indicates that 30 mg was used; however, in the Hi-C extraction section, it is indicated that 20-50 mg of tissue was used to prepare the Hi-C library.

 3. Haplotype 2 is indicated only through summary statistics. The authors should clarify whether haplotype 2 was scaffolded with the same Hi-C data and explain why it remains classified as scaffolded-level despite having an N50 comparable to that of haplotype 1.

 4. Table 2 and the main text report 58 scaffolds for haplotype 1; however, Figure 6 reports 59 scaffolds. Is the mitochondrial contig included? This should be clarified in the figure legend.

 5. Explain why 3 different MultiQC versions were used in the analyses.

Are sufficient details of methods and materials provided to allow replication by others?

Yes

Is the rationale for creating the dataset(s) clearly described?

Yes

Are the datasets clearly presented in a useable and accessible format?

Yes

Are the protocols appropriate and is the work technically sound?

Yes

Reviewer Expertise:

Whole genome sequencing, Whole transcriptome analysis, Shotgun metagenomics, Metabarcoding, Marine genomics

I confirm that I have read this submission and believe that I have an appropriate level of expertise to confirm that it is of an acceptable scientific standard.

# Reviewer report: Reviewer response for version 1

**DOI:** 10.21956/wellcomeopenres.29518.r161406

**Published:** 2026-08-03

**Authors:** Xue Qing

**Affiliations:** 1 Nanjing Agricultural University, Nanjing, China

This manuscript presents a chromosome-level, haplotype-resolved genome assembly of the geometrid moth Rheumaptera hastata. In general its already in good shape to be indexed. I just have few very minor comments:

 1. Species in Geometridae is difficult to identify. As a high quality reference genome, it would be nice if author can provide morphological voucher images for the taxonomic important character.  This data will be very valuable for further intra-specific variation/population genomic studies.

 2. it is not very clear for me if DNA and RNA were extracted from same individuals?

 3. it would be nice if author can also provide information on haplotype 2, e.g. assembly parameters, Hi-C map, quality assessment.

Are sufficient details of methods and materials provided to allow replication by others?

Yes

Is the rationale for creating the dataset(s) clearly described?

Yes

Are the datasets clearly presented in a useable and accessible format?

Yes

Are the protocols appropriate and is the work technically sound?

Yes

Reviewer Expertise:

genomics, evolution, invertbrates, zoology

I confirm that I have read this submission and believe that I have an appropriate level of expertise to confirm that it is of an acceptable scientific standard.
